# Manuka Honey Exerts Antioxidant and Anti-Inflammatory Activities That Promote Healing of Acetic Acid-Induced Gastric Ulcer in Rats

**DOI:** 10.1155/2017/5413917

**Published:** 2017-01-19

**Authors:** Saad B. Almasaudi, Aymn T. Abbas, Rashad R. Al-Hindi, Nagla A. El-Shitany, Umama A. Abdel-dayem, Soad S. Ali, Rasha M. Saleh, Soad K. Al Jaouni, Mohammad Amjad Kamal, Steve M. Harakeh

**Affiliations:** ^1^Biology Department, Faculty of Science, King Abdulaziz University, Jeddah 21589, Saudi Arabia; ^2^Special Infectious Agents Unit, King Fahd Medical Research Center, King Abdulaziz University, Jeddah 21589, Saudi Arabia; ^3^Biotechnology Research Laboratories, Gastroenterology Surgery Center, Mansoura University, Mansoura 35516, Egypt; ^4^Department of Pharmacology and Toxicology, Faculty of Pharmacy, King Abdulaziz University, Jeddah 21589, Saudi Arabia; ^5^Department of Pharmacology and Toxicology, Faculty of Pharmacy, Tanta University, Tanta 31111, Egypt; ^6^Animal Facility Unit, King Fahd Medical Research Center, King Abdulaziz University, Jeddah 21589, Saudi Arabia; ^7^Anatomy Department (Cytology and Histology), Faculty of Medicine, King Abdulaziz University, Jeddah 21589, Saudi Arabia; ^8^Department of Animal Physiology, Faculty of Veterinary Medicine, Mansoura University, Mansoura 35516, Egypt; ^9^Department of Hematology and Yousef Abdulatif Jameel Chair of Prophetic Medicine Application, Faculty of Medicine, King Abdulaziz University, Jeddah 21589, Saudi Arabia; ^10^King Fahd Medical Research Center, King Abdulaziz University, Jeddah 21589, Saudi Arabia; ^11^Yousef Abdulatif Jameel Chair of Prophetic Medicine Application, Faculty of Medicine, King Abdulaziz University, Jeddah 21589, Saudi Arabia; ^12^Special Infectious Agents Unit, King Fahd Medical Research Center, Yousef Abdullatif Jameel Chair of Prophetic Medicine Application, King Abdulaziz University, Jeddah 21589, Saudi Arabia

## Abstract

Gastric ulcers are a major problem worldwide with no effective treatment. The objective of this study was to evaluate the use of manuka honey in the treatment of acetic acid-induced chronic gastric ulcers in rats. Different groups of rats were treated with three different concentrations of honey. Stomachs were checked macroscopically for ulcerative lesions in the glandular mucosa and microscopically for histopathological alterations. Treatment with manuka honey significantly reduced the ulcer index and maintained the glycoprotein content. It also reduced the mucosal myeloperoxidase activity, lipid peroxidation (MDA), and the inflammatory cytokines (TNF-*α*, IL-1*β*, and IL-6) as compared to untreated control group. In addition, honey-treated groups showed significant increase in enzymatic (GPx and SOD) and nonenzymatic (GSH) antioxidants besides levels of the anti-inflammatory cytokine IL-10. Flow cytometry studies showed that treatment of animals with manuka honey has normalized cell cycle distribution and significantly lowered apoptosis in gastric mucosa. In conclusion, the results indicated that manuka honey is effective in the treatment of chronic ulcer and preservation of mucosal glycoproteins. Its effects are due to its antioxidant and anti-inflammatory properties that resulted in a significant reduction of the gastric mucosal MDA, TNF-*α*, IL-1*β*, and IL-6 and caused an elevation in IL-10 levels.

## 1. Introduction

Gastric ulcers develop inside the stomach, affect many people worldwide, and represent discontinuity in the gastric mucosal penetration through the muscularis mucosa [1]. This type of ulcer results from the imbalance between two known factors: aggressive factors (physical, chemical, or psychological) in the lumen and protective ones. The aggressive factors include acid, pepsin,* Helicobacter pylori*, stress, alcohol, administration of nonsteroidal anti-inflammatory drugs, and protective mechanisms which are present in the duodenal mucosa and include mucus, bicarbonate, prostaglandin, blood flow, the antioxidant system, sulfhydryl compounds, nitric oxide, and cell proliferation [1–3]. During the ulcer healing process, the eradication of* Helicobacter pylori* or control of gastric acid secretion was not sufficient to prevent recurrence of gastric ulcer [4–6]. A lot of research has been conducted and acquired knowledge over the years regarding the development of gastric ulcer. This resulted in the development of a wide spectrum of drugs for its treatment, like proton pump inhibitors, antacids, anticholinergics, and histamine receptor antagonists [7]. However, all the current therapies are not always effective, have adverse side effects, and are expensive. For this reason, identifying new potentially agents through natural sources is still essential for more effective and safe antiulcer therapy [8, 9].

Bees belonging to the species* Apis mellifera* collect the nectar from different flowers and convert it to honey. It has a density of around 1.36 g/mL (about 36% more dense than water) [10]. The medicinal application of honey in the treatment of ulcers was originally documented by the Sumerians and goes, as far, back as 2,100–2,000 BC [11]. Honey is effective in the treatment of a broad range of wound types including, but not limited to, burns, scratches, diabetic, malignant, leprosy, fistulas, leg ulcers, traumatic, boils cervical varicose ulcers, amputation burst abdominal wound septic and surgical wounds, cracked nipples, and wounds of the abdominal wall and perineum [12]. Natural honey is composed of around 82% carbohydrates, water, phytochemicals, proteins, minerals, and antioxidants. It is likely thought that the minor ingredients are likely to be responsible for differentiating among the various types of honey and for their medicinal and biological potential [13]. The sugars in honey include in a descending order the following: fructose (38.2%) and glucose (31.2%), sucrose (0.7%–1%), and disaccharides (approximately 9%) some trisaccharides and higher saccharides [14, 15]. Two important innovative commercial types of honey available on the market include manuka honey [16] and Surgihoney [17] and have been known for their effectiveness in wound management. In a previous study, we have demonstrated a gastroprotective effect of manuka honey against gastric lesions induced by ethanol [18]. In this study, we evaluate for the first time the gastric curative effects of manuka honey in rat model with acetic acid-induced chronic gastric ulcer. The underlying mechanism of such an effect is also elucidated.

## 2. Material and Methods

### 2.1. Animals

Sprague-Dawley male eight-week-old rats, weighing between 220 and 240 g, were used. The animals were housed for 1 week at a temperature of 24 ± 1°C and a 55 ± 5% relative humidity. They were reared on a standard laboratory diet and tap water ad libitum. The rats were deprived from food 24 hours prior to the experiment; during this period, animals were kept in cages with raised floors of wide mesh to prevent coprophagy while being allowed access to water ad libitum. Rats were handled following the animal care guideline set by our university. The experimental protocol was approved by Research Ethics Committee at King Fahd Medical Research Center.

### 2.2. Acetic Acid-Induced Gastric Ulcer and Treatment

Ulcer induction was achieved as described elsewhere [19]. Under anesthesia, laparotomy was performed on all animals through a midline-epigastric incision. The stomach was firstly exposed and then was injected with 0.05 mL (v/v) of a 30% acetic acid solution into the subserosal layer in the glandular part of the anterior wall. After that, the stomach was soaked in a bath of saline in order to prevent any adherence to the external surface of the ulcerated region. The abdomen then was closed afterwards to allow the rats to feed normally. Two days after surgery, all rats were randomly divided into six groups each consisting of 6 animals: (1) a SHAM control group which underwent the surgical procedure of ulcer induction with the application of saline instead of acetic acid; (2) control group: acetic acid ulcer induced group; (3) positive control group: acetic acid + ranitidine treated group (ranitidine at the dose of 30 mg/kg) [20]; (4) control group + low dose group of manuka honey (0.625 g/kg); (5) control group + medium dose group of manuka honey (1.25 g/kg); (6) control group + high dose group of manuka (2.5 g/kg). Treatment of rats was carried out two days after the induction of ulcer by gavage once a day for a period of 10 consecutive days. One day after the last treatment, the animals were sacrificed by cervical dislocation carried out under humane conditions and after the animals being anesthetized, the stomachs were removed and the mucosal damage was assessed according to the following: edema (1 point), hyperemia (1 point), petechiae (light, moderate, and intense with 1, 2, and 3 points, resp.), hemorrhagic lesion (3 points), ulcers (not perforated and perforated, 1 point/mm^2^ and 2 points/mm^2^, resp.), and thickening of the ulcer (1 point/mm^2^) [21].

(1) Ulcer  inhibition  rate=Control  ulcer  index−Test  (ulcer  index) Control  (ulcer  index)×100%.

Ulcer inhibition rate was expressed as previously described [22].

### 2.3. Pathological Effects on Gastric Tissue

Paraformaldehyde (4%) solution was used in order to fix the gastric tissues. This was followed by dehydrating the tissue samples with alcohol and xylene and later embedding them in paraffin for sectioning. 5 *μ*m thick sections were affixed onto slides, deparaffinized, and stained using hematoxylin and eosin (H&E). Light microscopy was used for the general histopathology examination.

### 2.4. Periodic Acid Schiff Staining for Determination of Mucin Content

The histochemical assay for the determination of mucin was performed as described earlier [19]. Samples were sectioned and placed on slide. These slides were then deparaffinized, rehydrated, oxidized (0.5% periodic acid for 5 min), and washed with distilled water. Then slides were stained with Schiff's reagent for 20 min, followed by washing the sections with sulfurous water (three times for 2 min) and in tap water for 10 min. Finally, the sections were counterstained with hematoxylin for 20 seconds and dehydrated.

### 2.5. Assessment of Gastric Mucosa Myeloperoxidase (MPO) Enzyme Activity

MPO was determined in gastric homogenates according to the method of Grisham et al. [20]. A portion of the stomach (100 mg) was homogenized in 10 volumes of ice cold potassium phosphate buffer (pH 7.4). The homogenate was centrifuged at 20,000 ×g for 20 min at 4°C. The pellets were then collected and were homogenized in 10 volumes of ice cold 50 mM potassium phosphate buffer (pH 6) containing 0.5% hexadecyl-trimethyl ammonium bromide (HTAB) and 10 mM EDTA. An aliquot of the homogenate (100 *μ*L) was removed and added to a 1 mL reaction volume containing 80 mM potassium phosphate buffer (pH 5.4), 0.5% HETAB, and 1.6 mM tetramethyl benzidine. The mixture was warmed to 37°C and then 100 *μ*L of 0.3 mM H_2_O_2_ added. The rate of change in absorbance was then measured at 655 nm. The MPO activity was expressed as U/mg tissue.

### 2.6. Sample Preparation for Antioxidant Analysis

Another portion of the stomach samples were homogenized in a solution of 2% Triton X-100 containing 0.32 M sucrose solution for SOD determination. Additional portions of the stomach were homogenized in 50 Mm potassium phosphate pH 7.5 and 1 Mm EDTA for determination of MDA, GSH, GPx, and CAT. The resulting homogenates were sonicated twice for 30 s intervals at 4°C and then centrifuged at 1800*g* for 10 min at 4°C [23].

### 2.7. Determination of Reduced Glutathione (GSH)

GSH was determined according to the method of Ellman [24] in stomach homogenates using kit from Biodiagnostic, Egypt. The GSH content of the tissue GSH was expressed as nmol/g tissue.

### 2.8. Determination of Lipid Peroxide (Measured as MDA)

The concentration of MDA was assayed in the stomach homogenates using kits from Biodiagnostic, Egypt. Based on the method of Uchiyama and Mihara [25], tissue MDA content was measured by taking two optical density measurements at two wavelengths (535 nm and 525 nm) and determining the difference between them. The distinct tissue MDA content was expressed as nmol/g tissue.

### 2.9. Determination of Glutathione Peroxidase (GPx)

This was done also on stomach homogenates using kits marketed by Biodiagnostic, Egypt, and based on what has been reported in the literature [26]. GPx activity was expressed in mU/g tissue.

### 2.10. Determination of Superoxide Dismutase (SOD)

The activity of SOD was done on stomach homogenates using kits provided by Biodiagnostic, Egypt; as described by others [27], SOD activity was expressed in U/g tissue.

### 2.11. Determination of Catalase (CAT)

The activity of CAT was assayed on stomach homogenates according to Aebi [28] using kit from Biodiagnostic, Egypt. CAT activity was expressed in U/g tissue.

### 2.12. Measurement of Tumor Necrosis Factor-*α* (TNF-*α*), Interleukin-1*β* (IL-1*β*), IL-6, and IL-10

ELISA kits (Assaypro, USA) were used for measurement of TNF-*α*, IL-1*β*, and IL-6 concentrations in stomach homogenate, while an ELISA kit (Novex, USA) was used for measurement of IL-10 concentrations in stomach homogenate. Cytokine concentrations were calculated using standard purified recombinant cytokines.

### 2.13. Analysis of Apoptosis and DNA-Cell Cycle by Flow Cytometry

Cell suspensions from specimens were collected from the stomach mucosa of rats treated with honey and from the control were treated with 0.1% Triton X-100 to create pores in cell membrane to allow the penetration of Propidium Iodide stain into the cell. The cells were washed with PBS at 37°C for 30 min in the dark and stained with a Propidium Iodide solution (50 *μ*g/mL Propidium Iodide and 50 *μ*g/mL RNase) (Sigma-Aldrich, Munich, Germany). Cell clumps of stained cells were removed by passing them through a nylon mesh sieve. Analysis was done using flow cytometry (BD FACSCalibur, San Jose, USA). Data collection and analysis was done using the CellQuest (BD, San Jose, USA) and ModFit Lt (Verity Software House Inc., Topsham, ME, USA) software.

Doublet discrimination was used to analyze the samples. This will allow the distinction between the signals originating from one nucleus versus two or more aggregated nuclei. Single nuclei will only be considered for the computer analysis. For each sample, data for 20,000 events were collected. The analysis of apoptosis was performed by determining hypodiploidy (sub-G1 peak) as previously described [29].

### 2.14. Statistical Analysis

All results were shown as mean ± SD. Data was entered using Statistics software SPSS 22. One-way analysis of variance (ANOVA) test was used to analyze the data. Statistical differences of *P* of <0.05 were considered to be significant.

## 3. Results

### 3.1. Effect of Manuka Honey on Ulcer Index

In the ulcer control group, the subserosal layer of the glandular part of the anterior stomach wall showed a significant increase in the gastric lesion index as compared to the SHAM value (*P* = 0.000). The administration of low concentrations of honey did not produce a significant reduction of the ulcer index and in the ulcer inhibition rate. However, a significant decrease in the gastric lesion index as well as in the ulcer inhibition rate was noted when rats were treated with 2.5 gm/kg or ranitidine (Table 1).

#### 3.1.1. Macroscopic Examination

**Figure 1:**
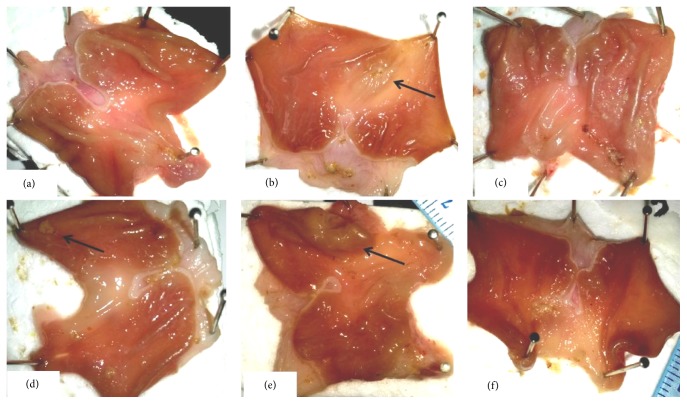
Effect of manuka honey on the regeneration of gastric mucosa examined in acetic acid-induced gastric ulceration in rats (gross examination). The images represent macroscopic photograph of the (a) SHAM, (b) ulcer control group, (c) positive control: ranitidine (30 mg/kg), (d) manuka honey (0.625 gm/kg), (e) manuka honey (1.25 gm/kg), and (f) manuka honey (2.5 gm/kg).

In the ulcer control group, the subserosal layer of the glandular part of the anterior stomach wall showed rounded gastric mucosal lesions (Figure 1(b)). The treatment of rats with low concentrations of manuka honey did not produce a significant healing ability (Figures 1(d) and 1(e)). However, when manuka honey was used at a high concentration of 2.5 gm/kg, it resulted in a significant healing effect of ulcer as compared to the SHAM group. The same was true in the case of the positive control ranitidine group (Figures 1(c) and 1(f)).

#### 3.1.2. Histopathological Changes of the Stomach Fundic Mucosa

**Figure 2:**
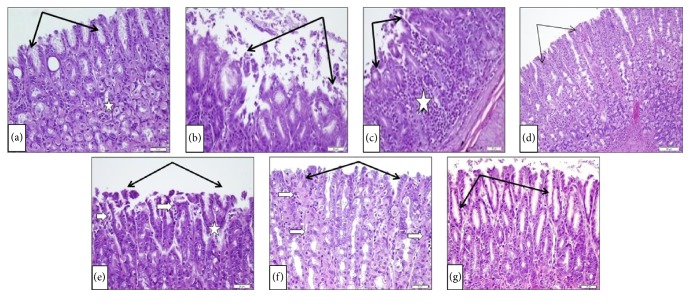
Effect of manuka honey on the histopathological changes of stomach fundic mucosa examined in acetic acid-induced gastric ulceration in rats (H&E ×20). (a) SHAM. (b and c) Ulcer control group. (d) The positive control (ranitidine). (e) Manuka honey (0.6 gm/kg); (f) manuka honey (1.25 gm/kg); (g) manuka honey (2.5 gm/kg).

As demonstrated in Figure 2, the SHAM group showed normal surface mucous columnar cells (black arrows) and intact glandular cells (star). This is in contrast to the ulcer control group (b and c) which showed disruption and desquamation of surface mucous epithelium (black arrows) with inflammatory cell infiltration (star). The positive control ranitidine (d) showed nearly normal surface cells. Using manuka honey (0.6 gm/kg) showed focal surface desquamation (black arrows), capillary congestion (white arrows), and necrosis (star) (e). Manuka honey at concentration of 1.25 gm/kg (f) demonstrated focal loss of mucous surface epithelium (black arrows) and mucosal inflammatory cell infiltrate (white arrows). In the case of manuka honey (2.5 gm/kg), marked proliferation and elongation of surface mucous cells extending deeper into mucosa (black arrows) (g) are shown.

#### 3.1.3. Effect of Manuka Honey on Glycoproteins

**Figure 3:**
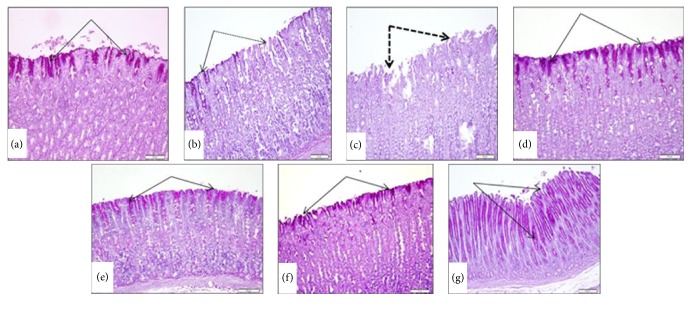
Effect of manuka honey on the gastric mucosal glycoprotein formation detected by PAS staining (PAS ×20). (a) SHAM showed high positive reaction in the surface mucous cells (black arrows). (b and c) Ulcer control group showed marked decrease (black arrows) or loss (dotted arrows) in PAS mucosal glycoprotein content of surface cells. (d) Positive control (ranitidine) showed marked preservation of mucopolysaccharides content of surface epithelium (arrows). (e) Manuka honey (0.6 gm/kg) showed moderate increase in PAS reacted glycoprotein. (f) Manuka honey (1.25 gm/kg) showed moderate increase in PAS reacted glycoprotein. (g) Manuka honey (2.5 gm/kg) showed marked increase in PAS reactive substance extending down along gastric glands (black arrows).

The subserosal layer of the glandular part of the anterior stomach wall showed either marked decrease or complete loss in gastric mucosal glycoprotein content of surface cells in the ulcer control group (Figures 3(b) and 3(c)). Treatment with manuka honey at 0.65 gm/kg or 1.25 gm/kg moderately increased PAS reacted glycoprotein (Figures 3(e) and 3(f)). Treatment of rats with either manuka honey (2.5 gm/kg) or ranitidine (30 mg/kg) showed marked preservation of the glycoprotein content of surface epithelium which may extend down along gastric glands (Figures 3(d) and 3(g)).

### 3.2. Assessment of Oxidative Stress Biomarkers

#### 3.2.1. Myeloperoxidase (MPO)

**Figure 4:**
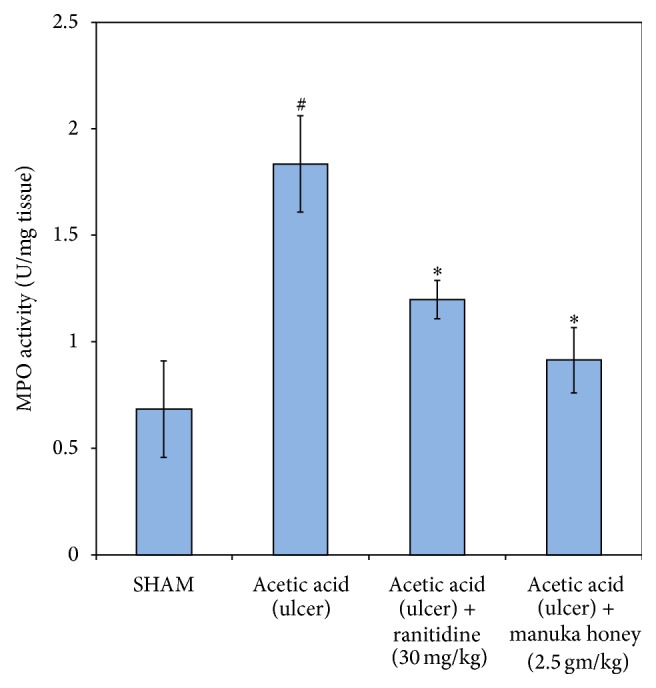
Effect of manuka honey (2.5 gm/kg) and ranitidine (30 mg/kg) on gastric mucosa myeloperoxidase (MPO) enzyme activity measured in acetic acid-induced gastric ulceration in rats. Each value is the mean ± SEM (*n* = 6). ^#^Significant versus SHAM (*P* ≤ 0.05). ^*∗*^Significant versus acetic acid (ulcer) (*P* ≤ 0.05).

There was a significant increase in the gastric mucosa MPO activity (170%) in the ulcer control group as compared to the SHAM group (*P* = 0.007). A significant decrease in the gastric mucosa MPO activity was noted in both the ranitidine and the honey (2.5 mg/kg) treated groups (50% and 35%, resp.) as compared to ulcer control group (*P* = 0.007  and  *P* = 0.026, resp.) (Figure 4).

#### 3.2.2. Glutathione (GSH)

**Figure 5:**
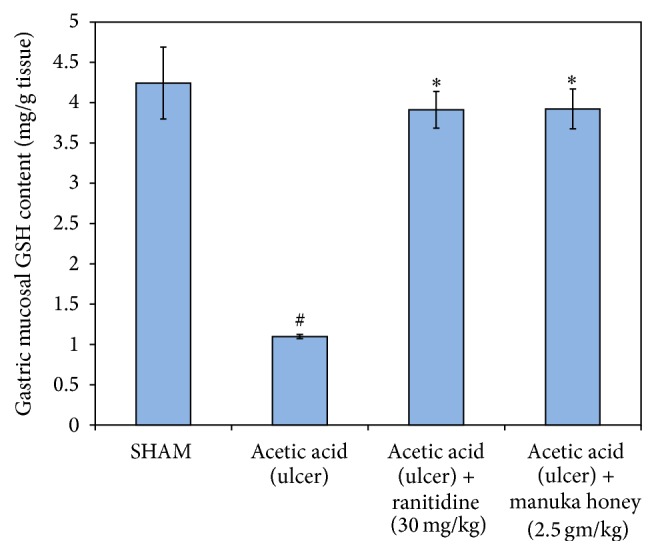
Effect of manuka honey (2.5 gm/kg) and ranitidine (30 mg/kg) on gastric mucosa reduced glutathione (GSH) content as compared to the ulcer control group and SHAM. Each value is the mean ± SEM (*n* = 6). ^#^Significant versus SHAM (*P* ≤ 0.05). ^*∗*^Significant versus acetic acid (ulcer) (*P* ≤ 0.05).

There was a significant reduction of the levels of GSH in the ulcer control group (74%) as compared to the SHAM value (*P* = 0.000). This significant decline was reversed upon treating the rats with either manuka honey (2.5 gm/kg) or ranitidine (30 mg/kg) resulting in a significant increase of gastric mucosal GSH content (~250%) as compared to ulcer control group (*P* = 0.000) (Figure 5).

#### 3.2.3. Malondialdehyde (MDA)

**Figure 6:**
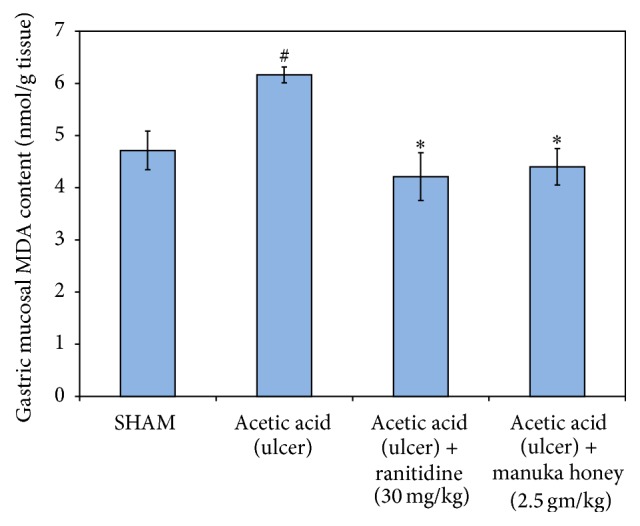
Effect of manuka honey (2.5 gm/kg) and ranitidine (30 mg/kg) on gastric mucosa malondialdehyde (MDA) content as compared to the ulcer control group and SHAM. Each value is the mean ± SEM (*n* = 6). ^#^Significant versus SHAM (*P* ≤ 0.05). ^*∗*^Significant versus acetic acid (ulcer) (*P* ≤ 0.05).

There was a significant increase in the gastric mucosa MDA levels (31%) in the ulcer control group in comparison to the SHAM group (*P* = 0.005). The treatment of rats with either manuka honey (2.5 gm/kg) or ranitidine (30 mg/kg) resulted in a significant decrease in the gastric mucosal MDA levels (~30%) as compared to ulcer control group (*P* = 0.001  and  *P* = 0.002, resp.) (Figure 6).

#### 3.2.4. Glutathione Peroxidase (GPX), Superoxide Dismutase (SOD), and Catalase (CAT) Enzyme Activities

In the ulcer control group, there was a significant decrease in the gastric mucosal GPx, SOD, and CAT activities (50%, 60%, and 28%, resp.) as compared to the SHAM value (*P* = 0.001, 0.003, and 0.000, resp.). Upon treating the rats with manuka honey (2.5 gm/kg), there was a significant increase in the gastric mucosa GPx, SOD, and CAT enzyme activities (78%, 109%, and 29%, resp.) as compared to ulcer control group (*P* = 0.001, 0.000, and 0.007, resp.). The treatment of rats with ranitidine (30 mg/kg) significantly increased gastric mucosa SOD and CAT enzyme activities (87% and 33%, resp.) as compared to ulcer control group (*P* = 0.000 and *P* = 0.023, resp.). However, ranitidine had no significant increase in GPx enzyme activity (19%) as compared to the ulcer control group (*P* = 0.238) (Table 2).

### 3.3. Effect of Manuka Honey on Gastric Mucosa Proinflammatory Cytokines: Tumor Necrosis Factor-Alpha (TNF-*α*), Interleukin-1 Beta (IL1-*β*), and IL-6

There was a significant increase in the gastric mucosal levels of TNF-*α*, IL1-*β*, and IL-6 (132%, 800%, and 53%, resp.) as compared to the SHAM group (*P* = 0.000, 0.000, and 0.005, resp.). The treatment of rats with manuka honey (2.5 gm/kg) significantly decreased gastric mucosal TNF-*α*, IL1-*β*, and IL-6 content (59%, 40%, and 20%, resp.) as compared to the ulcer control group (*P* = 0.000, 0.013, and 0.001, resp.). The same was true in the case of treatment with ranitidine where a significant decrease in gastric mucosal TNF-*α*, IL1-*β*, and IL-6 levels was noted (42%, 33%, and 23%, resp.) as compared to the ulcer control group (*P* = 0.000, 0.027, and 0.005, resp.) (Table 3).

**Figure 7:**
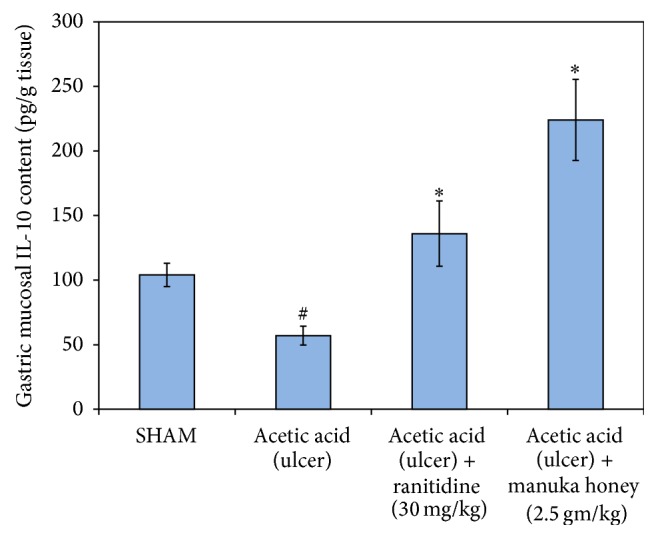
Effect of manuka honey (2.5 gm/kg) and ranitidine (30 mg/kg) on gastric mucosa interleukin-10 (IL-10) content as compared to the ulcer control group and SHAM. Each value is the mean ± SEM (*n* = 6). ^#^Significant versus SHAM (*P* ≤ 0.05). ^*∗*^Significant versus acetic acid (ulcer) (*P* ≤ 0.05).

*Effect of Manuka Honey on Gastric Mucosal Interleukin-10 (IL-10) Levels*. As was the case with other cytokines, the gastric mucosal levels of IL-10 were significant (45%) as compared to the SHAM group (*P* = 0.002). The treatment of rats with either manuka honey (2.5 gm/kg) or ranitidine (30 mg/kg) significantly increased gastric mucosal IL-10 levels (292% and 138%, resp.) as compared to the ulcer control group (*P* = 0.000 and 0.014, resp.) (Figure 7).

### 3.4. Effect of Manuka Honey on Cell Cycle Progression

**Figure 8:**
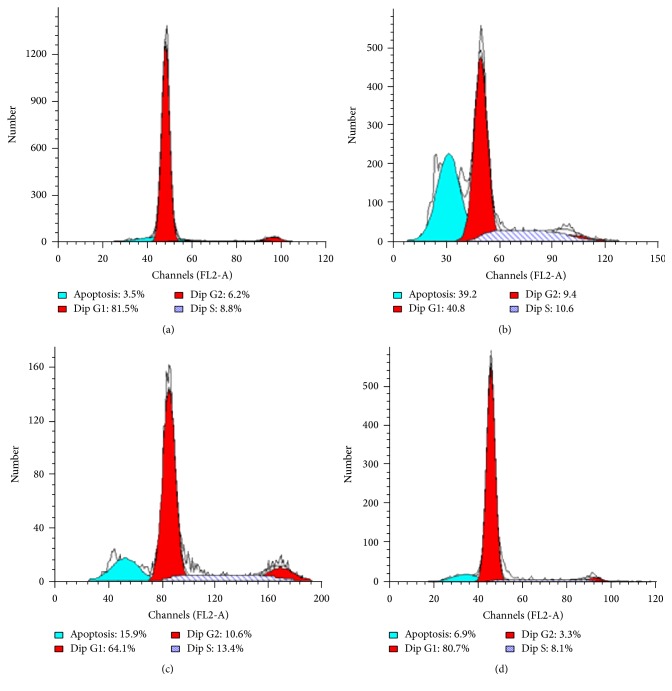
Schematic histograms of DNA-cell cycle analysis using flow cytometry. (a) represents stomach mucosa cells from SHAM group. (b) represents stomach mucosal cells from ulcer control group. (c) represents stomach mucosal cells from ulcer control group + ranitidine (30 mg/kg) group. (d) represents stomach mucosal cells from ulcer control group + manuka honey (2.5 gm/kg) group.

Significant increases in gastric mucosal apoptotic cell population (Sub-G1) and proliferation (S-phase) were seen in the ulcer control group as compared to the SHAM group (Figure 8). There was no change in the cell populations in the G_2_M phase in the ulcer control group as compared to the SHAM group (*P* = 0.585, resp.) (Table 4). Acetic acid in the ulcer control group significantly decreased G_1_ cell accumulation as compared with the SHAM group (*P* = 0.003) (Table 4). Treatments of rats with manuka honey (2.5 gm/kg) significantly decreased apoptotic cell population (Figure 8) and decreased sub-G_1_, S-phase, and G_2_M cell accumulation as compared to the control ulcer group (*P* = 0.004, 0.05, and 0.04, resp.) (Table 4). Also, manuka honey (2.5 gm/kg) significantly increased G_1_ cell accumulation as compared to the ulcer control group (*P* = 0.001) (Table 4). Treatments of rats with ranitidine (30 mg/kg) significantly decreased apoptotic cell population and G_2_M cell accumulation (Figure 8), as compared to the ulcer control group (*P* = 0.003) (Table 4). Ranitidine did not induce significant changes in cell accumulation in sub-G_1_, G_1_, and S-phase as compared to the ulcer control group (*P* = 0.572, 0.511, and 0.644, resp.) (Table 4).

## 4. Discussion

In the current study a significant increase was noted in the ulcer index and mean score in the acetic acid-induced ulcer group in comparison to the SHAM control group. The data obtained showed that treatment with manuka honey was safe to the animals used and resulted in zero mortalities. Manuka honey reversed the effects of acetic acid-induced oxidative injury and inflammation in the gastric mucosa and facilitated chronic ulcer healing. Such an effect is likely to be due to its constituents with biological activities including polyphenols such as flavonoids and phenolic acids and total water-soluble vitamins (vitamin B1, B2, B3, B9, and B12 and vitamin C) [30].

The acetic acid produces round, deep ulcers in the stomach and duodenum, resembling to a great extent human ulcer in terms of both pathological features and healing process. Such a model has been excessively used to study the pathophysiology and treatment of gastric ulcers and the underlying mechanisms involved in ulcer healing [31, 32].

It has been previously reported that acetic acid induces ulcer by penetrating the gastric mucosa and both the mucous membrane and submucous layers as well as the muscular layer. The ulcers produced by acetic acid become chronic within 2-3 days after ulcer initiation and may be completely treated within 2-3 weeks without the need for perforation or penetration to the surrounding organs [33, 34]. In addition, those ulcers can be treated with various antiulcer drugs [35]. We have previously demonstrated that manuka honey provided significant gastroprotective effects in acute gastric ulcer animal model [18]. The current data showed that manuka honey (2.5 mg/kg) had a healing potential comparable to ranitidine, which is a drug approved by the FDA and prescribed for the treatment of ulcers. Such a conclusion was based on macroscopic, histopathological, and flow cytometric data. These findings corroborate previous clinical published data on the use of manuka honey for chronic wounds healing [36–38]. One of these studies was observational and showed that manuka honey had positive antiulcer effects on 20 patients with spinal cord injuries and who suffered from chronic pressure ulcers (15 with grade III ulcers and 5 with grade IV ulcers) [36]. In the current study, manuka honey was able to reverse the decrease of the mucin-like glycoproteins, as observed by staining with PAS that are critical cytoprotective glycoproteins due to their mucus secretion activities. Such an action may be caused by phenols that are one of the main constituents of manuka honey [39]. Phenols stimulate the production of prostaglandin E2 (PGE2), which in turn produces mucus and, thus, results in providing protection of the gastrointestinal tract against injury [40].

The genesis of acetic acid-induced gastric lesions is a multifactorial process which starts mainly with the depletion of gastric wall mucous content [41]. Such a depletion is often associated with significant production of free radicals, causing damage to the cell and cellular membrane due to excessive oxidative stress [42]. The generation of reactive oxygen species (ROS), for example, superoxide anion, hydrogen peroxide, and hydroxyl radicals, may cause lipid peroxidation, especially in membranes, and results in tissue injury [9]. High levels of lipid peroxidation have been noticed in animals with induced ulcer. For this reason, the presence of MDA levels indicates more tissue damage due to the impairment of the antioxidants activities to deal with oxidative stress and with the handling of free radicals [43]. However, it has been reported that the first line of defense against oxidative damage caused by injury like ulcers involves the migration of free radical scavenging enzymes such as SOD, CAT, and GPx, to eliminate first O_2_ and H_2_O_2_ before forming harmful hydroxyl (OH^*∙*^) radical [44]. The present study revealed that there were significant increases in lipid peroxidation (MDA) and a reduction in the levels of GSH, GPX, SOD, and catalase in the untreated ulcer group compared with normal control group. This observation may emphasize the role of oxidative damage and ulcer induction, development, and/or maintenance. The data revealed that the oral administration of manuka honey as well as ranitidine interfered with the oxidative process through reduction of free radical level (MDA) and increased the levels of GSH, GPX, SOD, and catalase. These data suggest that manuka honey increases the activity of GPx to form GSH and augments the removal of reactive metabolites together with GSH. These data are in agreement with Henriques and colleagues [45] who reported that manuka honey possesses the most powerful antioxidant activities among all the different types of honey they tested and it was able to quench the introduced free hydroxyl radicals within 5 minutes after addition. Such a powerful antioxidant ability of manuka honey may be behind its potential to treat chronic inflammations, including ulcers.

Another possible mechanism by which manuka honey treats gastric ulcer may be due to inhibition of the proinflammatory cytokines: TNF-*α*, IL-1*β*, and IL-6. These cytokines are involved in production of acute inflammation [46], accompanied with neutrophil infiltration to the gastric mucosa [47], leading to gastric mucosal injury [48, 49]. It has been reported that manuka honey decreased the inflammatory response associated with ulcerative colitis, an inflammatory bowel disease characterized by an overexpression of inflammatory cells [50, 51]. The specific components that give manuka honey its activity are not yet determined [52]. However, it may be due to the presence of specific polyphenols, flavonoids, and caffeic acid phenethyl ester [53, 54]. The data obtained in this study revealed that IL-10 levels were significantly decreased in the ulcer control group. IL-10 is an anti-inflammatory cytokine and can limit tissue damage caused by inflammation [55]. Those results are similar to those reported by Eamlamnam et al. [55]. Such an effect may be due to the fact that when acetic acid induces gastric mucosal damage, T and B lymphocytes present in the submucosa beneath the damaged area and typically produce basal level of IL-10, become compromised, and fail to yield adequate levels of IL-10 [55]. Manuka honey elevated the IL-10 levels in the honey-treated group in comparison to the ulcer control group.

Inflammatory gastric diseases including gastric ulcer are commonly associated with increased epithelial proliferation [56]. However, chronic gastric ulcer is usually not associated with mucosal thickening, suggesting that the process of epithelial hyperproliferation is counterbalanced with cell losses, mainly through apoptosis [57]. In the current study, applying acetic acid to rats' gastric mucosa resulted in ulceration of the gastric mucosa that was associated with increased proliferation and apoptosis as indicated by DNA-flow cytometry analysis. These observations are consistent with previous reports on the induction of apoptosis in ulcerative gastric mucosa [58, 59]. On the other hand, administration of manuka honey significantly decreased percentage of apoptosis of gastric mucosa compared with that of untreated group. In addition, ulcer group treated with manuka honey showed significantly (*P* < 0.05) decreased proliferation as detected by DNA S-phase using flow cytometry. These data suggest that manuka honey counteracted the inflammatory effect of acetic acid on the gastric mucosa that resulted in the increase in proliferation and apoptosis.

## 5. Conclusion

This study demonstrated that manuka honey possesses a potent antiulcer activity, which may be due to its antioxidants abilities which result in reducing lipid peroxidation and interfering with the inflammatory process. The current study, therefore, adds to the long list of health benefits that are associated with consumption of honey and thus document its potency as a “functional food” that promotes better health.

## Figures and Tables

**Table 1:** Effect of different doses of manuka honey on gastric mucosal lesion index.

Treatment regimen	Gastric lesion index (mm^2^/stomach)	Ulcer inhibition rate (%)
SHAM	1.0 ± 0.17	—
Ulcer control group	15 ± 0.43^a^	—
Positive control (ranitidine)	8.0 ± 0.21^b^	47
Ulcer control group + manuka honey (0.625 gm/kg)	13 ± 0.60	13
Ulcer control group + manuka honey (1.25 gm/kg)	12 ± 0.63	20
Ulcer control group + manuka honey (2.5 gm/kg)	5.0 ± 0.31^b^	67

Data are mean ± SEM (*n* = 6).

^a^Significant versus SHAM (*P* ≤ 0.05).

^b^Significant versus acetic acid (ulcer) (*P* ≤ 0.05).

**Table 2:** Effect of manuka honey on gastric mucosa glutathione peroxidase (GPX), superoxide dismutase (SOD), and catalase (CAT) enzyme activities measured in acetic acid-induced gastric ulceration in rats.

Treatment regimen	GPX (U/g tissue)	SOD (U/mg tissue)	CAT (U/g tissue)
SHAM	1534 ± 113	257 ± 40	31 ± 0.9
Ulcer control group	767 ± 109^a^	101 ± 7^a^	23 ± 1.3^a^
Ulcer control group + ranitidine (30 mg/kg)	915 ± 46	188 ± 13^b^	31 ± 2.5^b^
Ulcer control group + manuka honey (2.5 gm/kg)	1368 ± 74^b^	210 ± 11^b^	30 ± 1.5^b^

Data are mean ± SEM (*n* = 6).

^a^Significant versus SHAM (*P* ≤ 0.05).

^b^Significant versus acetic acid (ulcer) (*P* ≤ 0.05).

**Table 3:** Effect of manuka honey (2.5 gm/kg) and ranitidine (30 mg/kg) on gastric mucosa tumor necrosis factor-alpha (TNF-*α*), interleukin-1 beta (IL1-*β*), and IL-6 content measured in acetic acid-induced gastric ulceration in rats.

Treatment regimen	TNF-*γ* (pg/mg tissue)	IL-1*β* (pg/mg tissue)	IL-6 (pg/mg tissue)
SHAM	274 ± 2.0	41 ± 0.5	113 ± 8
Ulcer control group	636 ± 30^a^	326 ± 41^a^	173 ± 6^a^
Ulcer control group + ranitidine (30 mg/kg)	367 ± 11^b^	217 ± 8^b^	132 ± 10^b^
Ulcer control group + manuka honey (2.5 gm/kg)	258 ± 13^b^	195 ± 13^b^	139 ± 5^b^

Data are mean ± SEM (*n* = 6).

^a^Significant versus SHAM (*P* ≤ 0.05).

^b^Significant versus acetic acid (ulcer) (*P* ≤ 0.05).

**Table 4:** Effect of manuka honey on cell cycle progression of the gastric mucosal cells.

Treatment regimen	Sub-G1%	G1%	S-phase%	G2M%
SHAM	4.39 ± 0.93	81.48 ± 2.46	6.70 ± 1.16	6.89 ± 1.04
Ulcer control group	24.39 ± 5.13^a^	57.82 ± 5.42^a^	10.40 ± 1.49^a^	7.93 ± 1.52^a^
Ulcer group + ranitidine (30 mg/kg)	20.36 ± 4.75^b^	64.01 ± 7.33^b^	8.69 ± 2.26^b^	1.95 ± 0.29^b^
Ulcer group + manuka honey (2.5 gm/kg)	4.91 ± 1.12^b^	84.83 ± 1.87^b^	3.78 ± 0.70^b^	4.13 ± 0.61^b^

Data are mean ± SEM (*n* = 6).

^a^Significant versus SHAM (*P* ≤ 0.05).

^b^Significant versus acetic acid (ulcer) (*P* ≤ 0.05).
